# Self-referential and social cognition in a case of autism and agenesis of the corpus callosum

**DOI:** 10.1186/2040-2392-3-14

**Published:** 2012-11-21

**Authors:** Michael V Lombardo, Bhismadev Chakrabarti, Meng-Chuan Lai, Simon Baron-Cohen

**Affiliations:** 1 Autism Research Centre, Department of Psychiatry, University of Cambridge, Douglas House, 18B Trumpington Road, Cambridge CB2 8AH, UK; 2 Centre for Integrative Neuroscience and Neurodynamics, School of Psychology and Clinical Language Sciences, University of Reading, Whiteknights RG6 6AL, UK

**Keywords:** Autism, Agenesis of the corpus callosum, Self, Theory of mind, Mentalizing, Social cognition

## Abstract

**Background:**

While models of autism spectrum conditions (ASC) are emerging at the genetic level of analysis, clear models at higher levels of analysis, such as neuroanatomy, are lacking. Here we examine agenesis of the corpus callosum (AgCC) as a model at the level of neuroanatomy that may be relevant for understanding self-referential and social-cognitive difficulties in ASC.

**Methods:**

We examined performance on a wide array of tests in self-referential and social-cognitive domains in a patient with both AgCC and a diagnosis of ASC. Tests included a depth-of-processing memory paradigm with self-referential and social-cognitive manipulations, self-report measures of self-consciousness, alexithymia, and empathy, as well as performance measures of first-person pronoun usage and mentalizing ability. The performance of the AgCC patient was compared to a group of individuals with ASC but without AgCC and with neurotypical controls. These comparison groups come from a prior study where group differences were apparent across many measures. We used bootstrapping to assess whether the AgCC patient exhibited scores that were within or outside the 95% bias-corrected and accelerated bootstrap confidence intervals observed in both comparison groups.

**Results:**

Within the depth-of-processing memory paradigm, the AgCC patient showed decreased memory sensitivity that was more extreme than both comparison groups across all conditions. The patient’s most pronounced difficulty on this task emerged in the social-cognitive domain related to information-processing about other people. The patient was similar to the ASC group in benefiting less from self-referential processing compared to the control group. Across a variety of other self-referential (i.e. alexithymia, private self-consciousness) and social-cognitive measures (i.e. self-reported imaginative and perspective-taking subscales of empathy, mentalizing), the AgCC patient also showed more extreme scores than those observed for both of the comparison groups. However, the AgCC patient scored within the range observed in the comparison groups on measures of first-person pronoun usage and self-reported affective empathy subscales.

**Conclusions:**

We conclude that AgCC co-occurring with a diagnosis of ASC may be a relevant model at the level of neuroanatomy for understanding mechanisms involved in self-referential and high-level social-cognitive difficulties in ASC.

## Background

Autism is a neurodevelopmental condition characterized by marked impairments in the domains of social interaction, language/communication, and pronounced repetitive stereotyped behaviors and/or restricted interests. The etiology of autism is complex as many genetic syndromes characterized by single-gene mutations (e.g., fragile X syndrome, Rett syndrome, tuberous sclerosis, Timothy syndrome) can result in a phenotype that meets the diagnostic criteria of autism (i.e. ‘syndromic autism’) [1]. Furthermore, research on non-syndromic forms of autism show that many simplex cases (up to 10% to 20%, but possibly more) possess *de novo* mutations in segments of DNA (i.e. deletions and/or duplications) presented as copy number variations [2-4]. This has resulted in the idea that there may be many types of autism arising from a complex combination of many different mechanisms.

In order to decompose some of this complexity, there is a need for clear models at a variety of levels of analysis. Autism genetics has already provided several useful models for understanding how genetic mechanisms linked to autism may have pathophysiological impact [5-8]. However, we currently lack clear models at higher levels of analysis such as at the level of neuroanatomy. In this paper, we focus on agenesis of the corpus callosum as one model at the level of neuroanatomy for dissecting aspects of self-referential and social-cognitive difficulties in autism. More specifically, we focus on areas within the self-referential domain related to the cognitive benefits of self-referential information processing (i.e. the self-reference effect in memory) as well as difficulties in emotional awareness (i.e. alexithymia), which are known to be more pronounced in higher-functioning adults with ASC. Within the social-cognitive domain, we focus on higher-level social-cognitive understanding of others (i.e. mentalizing, empathy), as well as memory for social agents; each of which have been shown to be sensitive to deficits in higher-functioning adults with ASC.

Agenesis of the corpus callosum (AgCC) is a congenital condition manifested in the failure to completely develop a corpus callosum; the largest white matter tract connecting the two cerebral hemispheres [9,10]. AgCC can present as a complete or partial absence of the CC. Complex AgCC occurs with the presence of other confounding brain abnormalities such as polymicrogyria, heterotopia, or schizencephaly, whereas isolated AgCC does not occur with such abnormalities. However, isolated AgCC commonly co-occurs with colpocephaly (i.e. dilation of the posterior section of the lateral ventricles and reduction of ipsilateral cortical association tracts) and Probst bundles [10]. The etiologies of AgCC are complex and likely reflect a multitude of routes important for fully establishing callosal connections (i.e. cellular proliferation and migration, axon guidance and growth, glial development, and midline patterning). Many genetic syndromes are associated with AgCC (e.g., X-linked lissencephaly, Mowat-Wilson syndrome, CRASH syndrome), and some of these syndromes have been associated with autism (i.e. *ARX* mutations leading to X-linked lissencephaly [11]). However, like autism, the majority of AgCC cases do not have an identifiable single genetic cause [10]. There are important clinical and neuroanatomical parallels between AgCC and autism that may suggest the role of atypical callosal development in understanding the emergence of phenotypes associated with autism.

First, one of the more replicable findings in neuroscience research on autism is marked atypicalities in the corpus callosum (CC). In a meta-analysis of volumetric MRI studies on the CC in autism, Frazier and Hardan [12] found that all subsections are consistently observed as reduced in volume. This result is further bolstered by longitudinal evidence for persistence of CC volumetric reduction at a 2-year follow-up examination [13]. In diffusion MRI work, several atypicalities in CC integrity, particularly with respect to reduced fractional anisotropy and increased mean diffusivity are reported [14-20]. Finally, a recent study using magnetization transfer imaging as an index of processes relevant to myelination found evidence for atypical myelination of the CC in autism [21]. The mounting evidence for atypical CC development in autism, particularly with respect to reduced size, potentially indicates that mechanisms involved in AgCC may also be important in autism.

Second, there are important clinical aspects that overlap between AgCC and autism. One of the most well known cases of such overlap is the case of the real ‘Rainman’, Kim Peek, who was diagnosed with autism well before later MRI scans determined he also had complete AgCC. Approximately 3% to 5% of individuals with neurodevelopmental conditions also have AgCC or CC hypoplasia [22,23], and this is of particular relevance for autism, where CC volume is notably reduced [12]. In addition, 8.5% of individuals with AgCC also have a specific diagnosis of autism [24]. However, this estimate may be conservative [9,25]. Badaruddin *et al*. [26] reported that in a sample of 61 two- to eleven-year-old children with AgCC, as many as 34% met criteria for at least one item in the domain of social impairment, 25% met criteria for at least one item in the communicative domain (with 51% showing difficulty in sustaining conversation), and up to 28% had a preoccupation with a specific interest. More recently, in 106 patients with AgCC, Lau *et al*. [27] found that 43% of children, 35% of adolescents, and 18% of adults score above cut-offs on the Autism Spectrum Quotient (AQ) that are typically used for screening for clinical diagnoses of autism spectrum conditions (ASCs).

Among the most prominent findings in the neuropsychological profile of AgCC are impairments in self-referential and social cognition [10]. Within the social-cognitive domain, many atypical findings in AgCC overlap with those seen in autism. Theory of mind or mentalizing impairments have long been known in autism [28,29], and these impairments can be helpful for explaining difficulties in pragmatic aspects of social communication [30]. Deficits in cognitive aspects of empathy [31-33] and on self-report measures of empathy [34-38] have been consistently reported in ASC. Affective components of empathy are spared in ASC [31,33], although self-report measures of this component are somewhat inconsistent. Some studies show decreases on subscales such as empathic concern [35,39] but increases on subscales such as personal distress [35]. The latter subscale is likely linked to comorbid anxiety traits in ASC [40]. Still other reports of self-reported affective empathy show no differences [36,38]. Given difficulties in ASC with self-referential cognitive processing, it is likely that the experimental studies on the topic comparing ASC to other groups with dissociable empathy deficits, such as individuals with psychopathic tendencies or conduct disorder [31,33], give the clearest indication of the empathy profile in ASC rather than relying solely on self-report measures.

Several studies now show that individuals with AgCC have marked impairments in pragmatic aspects of language processing, including comprehension of idioms, proverbs, vocal prosody, and non-literal interpretation of humorous statements [41-44]. Outside of pragmatic social communication difficulties, AgCC also shares social-cognitive deficits with those seen in autism. These difficulties extend into difficulty providing narratives that demonstrate understanding of social-emotional aspects of stories [45-47]. There are mixed reports regarding AgCC and ToM deficits. One study found deficits on the Reading the Mind in the Eyes test in some, but not all, AgCC patients [25]. However, other work on adolescents and adult patients did not reveal deficits on other advanced ToM stories and faux pas tests [48] known to be sensitive to deficits within autism [49,50]. While Symington *et al*. [48] did not find such advanced ToM deficits, they found difficulties in AgCC when asked to interpret videotaped social vignettes which indicated subtle deficits in emotion recognition, understanding sarcasm, and difficulty interpreting textual cues [48]. Finally, AgCC patients tend to underestimate emotional valence and intensity from negatively valenced pictures [51]. However, no studies exist in AgCC that comprehensively test components of empathy, making comparison with ASC difficult.

Prior work in the domain of self-referential cognition also indicates that difficulties in autism [35,52-54] may be shared by patients with AgCC. First, individuals with ASC show deficits across a wide range of areas where self-relevant information processing is critical, such as emotional understanding (e.g., alexithymia) [55], memory [35,56], introspection [57], pronoun usage [35,58], orienting to name [59,60], monitoring own intentions [61], remembering own false beliefs [62], attributing privileged access to self over close others [63] (for review see [52,53,56,58,64,65]). Within AgCC, one case study highlighted the possibility that alexithymia may be more pronounced [66]. This observation is similar to other reports on alexithymia hypothesizing that one aspect of its neurophysiological basis is reduced interhemispheric transfer [66-69]. Parental reports indicate that patients with AgCC show poor personal insight [10]. Also indicative of a potential lack of insight is the effect that parental ratings tend to be higher than self-reported ratings on the AQ [27]. Analysis of narrative content indicates that patients with AgCC tend to use more first-person pronouns than comparison groups [46]. Finally, Brown and Paul [70] suggest that high scores on the Minnesota Multiphasic Personality Inventory Lie scale (without elevations in other scales) may be indicative of poor self-awareness in the two AgCC cases they tested.

Despite the evidence reviewed here with regard to self-referential and social cognition, much work is still needed. At present, very few studies on AgCC exist (e.g., [48]) that assess social-cognitive abilities known to be clearly atypical in autism. In addition, even less research in AgCC specifically tests known self-referential atypicalities in autism. In this study, we add to this literature by using a battery that measures processes such as empathy, mentalizing, alexithymia, self-referential memory biases, private self-consciousness, and implicit self-focused attention. We also attempt to go further by assessing a case with both partial AgCC and autism in comparison to both neurotypical controls and cases of autism without AgCC. By comparing this single case to the two groups, we examined the extent to which aspects of his self-referential and social-cognitive profile overlap with the pattern found in autism and also identified aspects which may be even more atypical than those seen in autism. From the viewpoint that individuals with autism typically have smaller CCs than controls, AgCC could be construed as an extreme of this profile. Therefore, highlighting areas in AgCC that are even more extreme compared to the autism group may provide some indication that the CC is critical for such aspects of self-referential and social cognition.

## Methods

### Patient AG

The patient reported in this study has diagnoses of partial AgCC and Asperger syndrome (AS) and will be referred to from here on as patient AG. AG was a participant of a larger multi-centre MRI study of ASC: the MRC AIMS Consortium study [54,71-78]. AG gave informed consent for the study in accordance with ethics approval from the National Research Ethics Committee, Suffolk, England. Upon AG’s recruitment, we considered him as having only a clinical diagnosis of AS, but later his AgCC was incidentally found by neuroradiological examination of his brain structural MRI scans. In keeping with the MRC AIMS protocol, both the Autism Diagnostic Interview–Revised (ADI–R) and the Autism Diagnostic Observation Schedule (ADOS) were conducted before MRI scanning to confirm clinical diagnosis of ASC. AG scored above the cut-offs on both the ADI–R and ADOS (see Table 1 for scores and demographic information). In accordance with the recruitment criteria for the MRC AIMS Consortium, AG did not have any other comorbid medical (e.g., epilepsy) or neuropsychiatric conditions (e.g., attention-deficit/hyperactivity disorder, obsessive-compulsive disorder). AG’s level of adaptive functioning was not rigorously assessed with any standardized measures, but parental report indicated limited living independence (i.e. living with parents, no full-time employment). 

**Table 1:** **Participant characteristics**^**a**^

	**Control**	**ASC**	**AG**
*N* (Sex)	30 (23 M, 7 F)	30 (23 M, 7 F)	1 (M)
Age	29.93 (7.83)	29.13 (7.40)	29
VIQ	116.47 (8.65)	116.13 (12.81)	87
PIQ	114.43 (10.08)	114.17 (14.21)	87
FIQ	117.10 (8.65)	117.23 (13.11)	85
AQ	16.50 (6.38)	33.93 (7.89)	36
ADOS Comm-Soc	-	-	7
ADI-Social	-	-	22
ADI-Comm	-	-	12
ADI-Rep	-	-	10

^a^Mean and standard deviations (in parentheses) are given for the control and ASC groups. AG’s performance is given in the third column. ADOS and ADI scores reflect diagnostic algorithm scores. ASC, autism spectrum condition; VIQ, verbal IQ; PIQ, performance IQ; FIQ, full-scale IQ; ADOS, Autism Diagnostic Observation Schedule; ADI, Autism Diagnostic Interview; Comm, communication.

**Figure 1:**
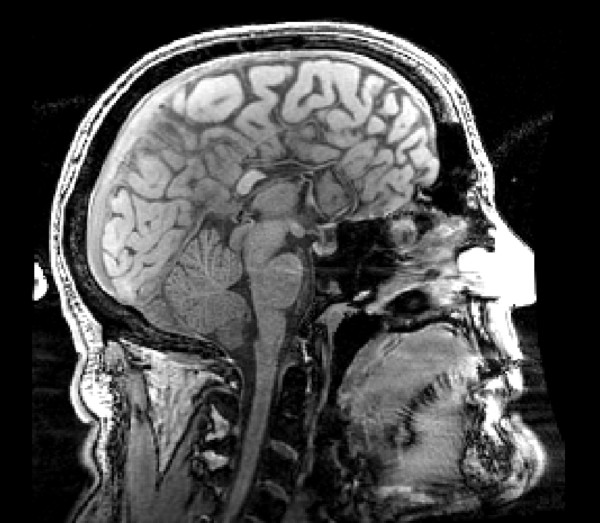
**Structural MRI scan of patient AG.** AG has partial agenesis of the corpus callosum (AgCC) and a clinical diagnosis of an autism spectrum condition (ASC). AG also showed signs of colpocephaly; dilation of the posterior aspect of the lateral ventricles.

After completing the MRI scan (see Figure 1), it was immediately apparent that AG lacked almost all of the CC. A partial segment of the CC was intact in the position of what would typically be the splenium and/or isthmus. However, given the variability in positioning and connectivity of partial segments of the CC in partial AgCC, we cannot say with certainty that the splenium/isthmus was the subsection that was intact [79]. The anterior commissure was visible and intact. However, Probst bundles were visibly emanating in an anterior direction from the intact section of the CC. There were also signs of colpocephaly (i.e. dilation of the posterior aspect of the lateral ventricles.) There were no signs of polymicrogyria or heterotopic sigmoid bundles connecting occipital and frontal cortex. An independent radiologist confirmed these observations and converged upon the same diagnosis. AG’s general practitioner was informed immediately in accordance with standard operating procedures for incidental MRI findings. 

### Comparison groups

In order to assess self-referential and social cognition in AG, we used the same battery of tests reported in our previous paper on autism [35]. In that paper, we found across a variety of different measures a robust pattern of co-existing deficits in autism within the domains of self-referential and social-cognitive processing. Because we wanted to compare AG’s presentation to these patterns in autism, the control and ASC groups from this previous study [35] were used to provide comparison data to AG. All control and ASC participants in the prior study gave informed consent in accordance with ethics approval from the University of Cambridge Psychology Research Ethics Committee. There were 23 males and 7 females ages 19 to 45 years who had a formal diagnosis based on DSM-IV or ICD-10 criteria of either high-functioning autism (HFA; *n* = 4) or AS (*n* = 26). Controls consisted of 23 males and 7 females who were pairwise-matched with the ASC participants on age and sex and had no known psychiatric, developmental, or neurological disorders. All participants completed the AQ [80], and the ASC group scored higher than controls (*P* < 0.001). All participants scored within the normal range on the Wechsler Abbreviated Scale of Intelligence (WASI) [81], and there were no statistically significant group differences on verbal, performance, or full-scale IQ (all *P* > 0.90). See Table 1 for participant characteristics.

## Procedure and measures

### Self-reference effect in memory (SRE) paradigm

The main experimental paradigm for testing both self-referential and social-cognitive information processing was the self-reference effect in memory paradigm (SRE). This paradigm allows participants to encode trait adjectives by judging them in relation to how descriptive they are of themselves or other people. Other encoding manipulations in this paradigm might involve semantic encoding manipulations or judgments based on basic linguistic characteristics. After encoding, participants are given a surprise memory test, and the typical trend is for heightened memory sensitivity for information previously encoded in relation to oneself when compared to other encoding conditions (i.e. thinking about others, semantic manipulations, linguistic characteristics). There is generally a gradient in memory performance across this paradigm, with the best memory for self-relevant information, followed by memory for others, semantic manipulations, and worst memory for shallow encoding based on basic linguistic characteristics. The SRE paradigm is a ‘depth-of-processing’ paradigm [82] because it is thought that the ‘deepest’ level of encoding in memory tends to be around structures that can easily organize and elaborate on information and the self possesses such properties that allow for deep organization and elaboration of information [83-85].

For the current study, in the encoding phase, participants judged trait adjectives in one of four ways. In the ‘self’ condition, participants judged how descriptive the adjective was of themselves. In the ‘similar close other’ condition, adjectives were judged on how descriptive they were of their best friend. In the ‘dissimilar non-close other’ condition, participants judged whether the adjective was descriptive of Harry Potter [86]. All of these judgments were made on a 6-point scale, where 1 indicated ‘not at all descriptive’ and 6 indicated ‘very descriptive’. Finally, in the non-social control condition, participants judged how many syllables each adjective contained (from 1 to 6).

Each condition had 30 trials, and all were presented in pseudorandom order. All adjectives were drawn from a previously validated and widely used set of trait adjectives [87]. Half the adjectives in each condition were positively valenced (e.g., inventive), and the other half were negatively valenced (e.g., messy). Among all conditions, there were no differences in the number of characters, syllables, valence, or frequency of the adjectives. After encoding, there was a 30-minute delay before the retrieval phase. Participants were completely unaware of the subsequent recognition memory task to follow. During this delay, participants completed the performance section of the WASI. These tasks were nonverbal and were administered to keep the participant occupied during the delay period.

After the delay, participants were given a surprise recognition memory test. All 120 adjectives from encoding and 120 new distracter adjectives were presented in pseudorandom order. Participants judged their confidence in whether the adjective was ‘old’ or ‘new’. Confidence judgments were made on a 1 to 6 scale, where 6 was ‘definitely OLD’ and 4 was ‘OLD, but kind of unsure’. Conversely, a 1 indicated that they were ‘definitely NEW’ and 3 was ‘NEW, but kind of unsure’. This 6-point scale was used to force participants to make finer-grained recognition judgments and also to investigate whether there were any differences in how each group used different confidence judgments. Because there were no group differences among judgments on each scale point within any of the conditions, we collapsed judgments 1 through 3 into ‘New” and 4 through 6 into ‘old’ judgments. Furthermore, because all of the data from the control and ASC comparison groups come from a previously published report, we refer to that paper for details on the manipulation checks to ensure that participants did perceive best friends as more similar and closer to self than Harry Potter (see Manipulation Check section [35]). Finally, the dependent variable from this paradigm was a standard measure of memory sensitivity (d′), formulated as the standardized score of correctly remembered words minus the standardized score of false alarms. Higher d′ scores indicate enhanced memory sensitivity.

### Additional self-referential and social-cognitive measures

In addition to the SRE paradigm, we included three other measures in the domain of self-referential cognition. These measures are included as a supplement to the self-referential memory sensitivity measure from the SRE paradigm and because they are theoretically relevant measures for testing more generalized deficits in self-referential processing in ASC [35]. If self-referential processing is atypical, then we expect to observe atypical performance across these additional measures as well as on the self-condition from the SRE paradigm. Self-report measures such as the Private Self-Consciousness Scale (PSCS) [88] and the Toronto Alexithymia Scale (TAS) [89] were used to measure explicit self-focused attention and emotional awareness. The TAS was further split into difficulty identifying feelings (DIF), difficulty describing feelings (DDF), and externally oriented thinking (EOT) subscales. On the TAS and all its subscales, higher scores indicate more alexithymic traits and can be interpreted as more impaired. We also included a performance measure of implicit self-focused attention called the Self-Focus Sentence Completion task [90]. In this task, participants were given sentence stems that included a self-reference (e.g., “I think…,” or “If I had my way…”). For each stem, we asked participants to complete the sentence in whatever way they liked. We computed an index of implicit self-focused attention (SFA) by automatically computing the percentage of first-person pronouns used to complete the stems (e.g., I, me, myself) using the Linguistic Inquiry and Word Count program [91]. This index has been used previously as a quantitative index of SFA [92-94]. Higher scores on this index indicate more implicit self-focused attention.

In addition to the two ‘other’ conditions within the SRE paradigm, we included further measures in the domain of ‘other-oriented’ social-cognitive processing. Again, similar to the rationale behind the additional self-referential measures, these measures were included as a supplement to the social-cognitive memory sensitivity measures observed on the SRE paradigm and because they are theoretically relevant measures for testing more generalized deficits in social-cognitive processing in ASC [35]. If social-cognitive processing is atypical, then we expect to observe atypical performance across these additional measures as well as on the Friend and Potter conditions of the SRE paradigm. Three self-report measures of empathy were used: the Empathy Quotient (EQ) [34], the Interpersonal Reactivity Index (IRI) [95], and the Emotional Contagion Scale (ECS) [96]. The IRI was further split into perspective-taking (IRI-PT), empathic concern (IRI-EC), fantasy (IRI-FS), and personal distress (IRI-PD) subscales. Finally, we included the ‘Reading the Mind in the Eyes’ test (RMET) as one advanced performance measure of mentalizing/theory of mind [32].

### Statistical analyses

To estimate similarities and differences between AG and the control and ASC comparison groups, bootstrapping (1 million resamples) [97] was used to estimate bias-corrected and accelerated 95% confidence intervals around the mean statistic from the control and ASC groups. AG’s performance was then compared to these bootstrap distributions. If AG’s performance was outside the bootstrap confidence intervals, we inferred that AG’s performance was significantly more extreme than performance on average within the comparison group.

Because AG had IQ scores below the comparison groups, further correlation analyses were run on the ASC group only (the one comparison group that partially overlaps with AG’s clinical presentation of ASC) in order to examine whether IQ, particularly verbal IQ (VIQ), was related to any of the dependent variables. For these analyses, we used robust regression [98] to estimate correlations with IQ that are robust to the influence of outlying data points.

## Results

Table 1 shows participant characteristics for patient AG and the comparison groups. AG was similar to the mean ages of the two comparison groups. On IQ scales, AG was approximately 2 standard deviations below the mean of the comparison groups. Given the IQ differences, for all further analyses, we ran robust regression within the ASC group to assess the correlation between VIQ and the dependent measures while being insensitive to outliers. Any significant correlations within the ASC group would signal potentially that AG’s performance might be influenced by lower IQ than the comparison groups.

**Figure 2:**
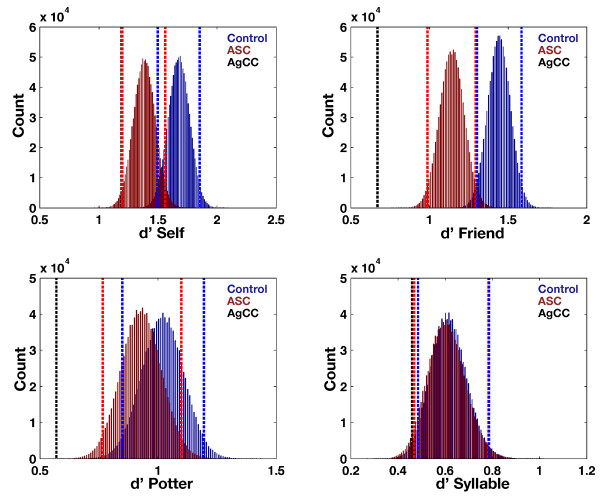
**Self-reference effect in memory (SRE) data.** This figure plots the bootstrap distributions of the mean along with the 95% bias-corrected and accelerated bootstrap confidence intervals (vertical dotted lines). The ASC group is depicted in red and the control group in blue. AG is depicted as the black dotted line. Abbreviations: d′, memory sensitivity; ASC, autism spectrum condition; AgCC, agenesis of the corpus callosum.

On the SRE paradigm, AG’s performance was significantly worse than both the ASC and control groups on all conditions, as noted by performance below the lower-bound 95% bootstrap confidence interval. The conditions with the largest deficits in performance were in social-cognitive conditions (Friend and Potter), whereas more subtle deficits were apparent across Self and Syllable conditions (see Figure 2 and Table 2). These results could not easily be explained simply by generalized slowed speed of processing in AgCC [99], because our patient responded with speed similar to, or in some cases faster than, both comparison groups across encoding and retrieval phases (see Additional file 1: Figure S1). Correlations between VIQ and memory sensitivity were assessed next to ascertain if AG’s performance might be explained by correlation between IQ and memory sensitivity in the ASC group. Here we found that correlations were positive for all conditions and were nominally significant (i.e. *P* < 0.05 but did not survive corrections for multiple comparisons) for VIQ in relation to Friend (*r* = 0.51, *P* = 0.0134) and Potter (*r* = 0.45, *P* = 0.0268) conditions (see Additional file 1: Figure S2). This potentially suggests that worse memory performance in AG can be partially explained by an IQ lower than the IQs in the comparison groups. 

**Table 2:** **Self-reference effect in memory (SRE) paradigm**^**a**^

	**Control**	**ASC**	**AG**
d′ Self	1.68 (1.50 to 1.85)	1.38 (1.20 to 1.56)	1.19
d′ Friend	1.44 (1.30 to 1.59)	1.14 (0.99 to 1.29)	0.67
d′ Potter	1.02 (0.85 to 1.20)	0.93 (0.77 to 1.10)	0.57
d′ Syllable	0.62 (0.49 to 0.79)	0.61 (0.47 to 0.78)	0.46

^a^Mean and 95% bootstrap bias-corrected and accelerated confidence intervals (in parentheses) are given for the control and ASC groups. AG’s performance is given in the third column. In each condition, AG’s performance was below the lower bound 95% confidence interval for both control and ASC groups. ASC, autism spectrum condition; d′, index of memory sensitivity.

**Figure 3:**
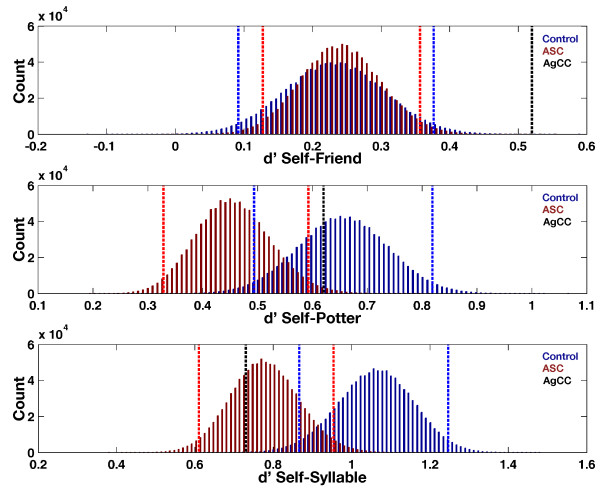
**Self-reference effect in memory difference-score measures.** This figure plots the bootstrap distributions of the mean difference-score of memory sensitivity measures on the SRE paradigm along with the 95% bias-corrected and accelerated bootstrap confidence intervals (vertical dotted lines). The top panel represents the self-reference effect in memory when Self-memory sensitivity is compared to Friend-memory sensitivity. The middle panel represents the self-reference effect in memory when Self-memory sensitivity is compared to Potter-memory sensitivity. The bottom panel represents the self-reference effect in memory when Self-memory sensitivity is compared to Syllable-memory sensitivity. The ASC group is depicted in red and the control group in blue. AG is depicted as the black dotted line. Abbreviations: d′, memory sensitivity; ASC, autism spectrum condition; AgCC, agenesis of the corpus callosum.

Next we computed measures of the self-reference effect in memory, which is operationalized as the difference-score between memory sensitivity for self- and other non-self-encoding conditions (see Figure 3) [35,83]. On this measure, an increasingly positive value means larger cognitive benefits for self-referential information processing. Given the descriptive statistics in Table 1, it is not surprising that we found that AgCC had an enhanced SRE difference-score compared to both the ASC and control groups for Self-Friend. However, this difference is driven primarily by the large drop-off in memory sensitivity in AG for the Friend and Potter conditions (relative to what is observed in the control and ASC groups). Given that the ASC and AgCC groups are predicted to have social-cognitive deficits, the social-cognitive conditions are not the best for using in such difference-scores as an assessment of whether one benefits from self-referential information processing. The reason here is that deficits in the social-cognitive domain can affect the difference-score, and, rather than it saying something about self-referential processing, it has more to say about social-cognitive deficits. Therefore, the Self-Friend and Self-Potter difference-scores do not provide an unambiguous measure of whether AG benefits from self-referential information processing. 

In contrast, the interpretation behind the Self-Syllable measure is different. Here the difference-score is not contaminated with effects due to both self-referential and social-cognitive processing and allows for a more unambiguous look at whether AG benefits from self-referential processing. AG shows relatively similar memory sensitivity for the Syllable-condition (see Table 1 and Figure 2) but more pronounced dropoff in the Self-condition, particularly with respect to the control group. Therefore, the Self-Syllable difference-score shows a smaller self-reference effect in memory for AG compared to the control group, but similar effect compared to the ASC group. This effect can be more unambiguously interpreted as a deficit for AG in cognitive benefits obtained from self-referential information processing because it is primarily driven by a dropoff in AG’s memory sensitivity for the Self-condition. This deficit is one that AG shares with the ASC group, but he cannot be considered an extreme of the ASC group.

**Figure 4:**
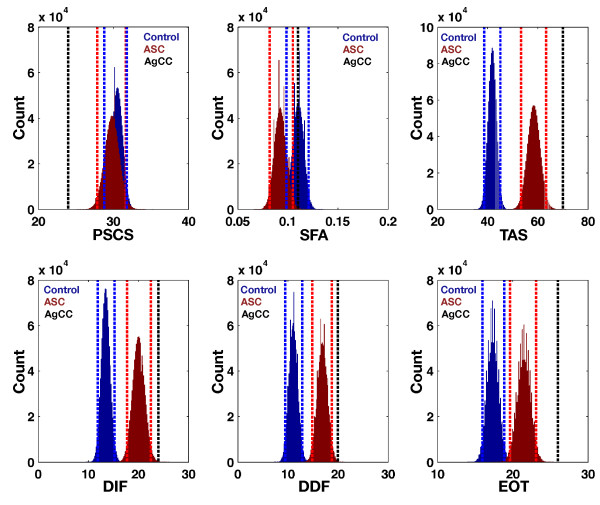
**Additional self-referential measures.** This figure plots the bootstrap distributions of the mean along with the 95% bias-corrected and accelerated bootstrap confidence intervals (vertical dotted lines). The ASC group is depicted in red and the control group in blue. AG is depicted as the black dotted line. Abbreviations: PSCS, Private Self-Consciousness Scale; SFA, self-focused attention index measured as the percentage of first person pronouns used in the Self-Focus Sentence Completion Task; TAS, Toronto Alexithymia Scale; DIF, Difficulty Identifying Feelings subscale of the TAS-20; DDF, Difficulty Describing Feelings subscale of the TAS-20; EOT, Externally Oriented Thinking subscale from the TAS-20; ASC, autism spectrum condition; AgCC, agenesis of the corpus callosum.

On additional self-referential measures, AG’s performance was also significantly worse on the TAS and all its subscales and reported less private self-consciousness on the PSCS. However, on the SFA measure, AG used significantly more first-person pronouns than the ASC group but was similar to the control group. Thus, his performance on this test is not in keeping with the slightly lower production of first-person pronouns observed in the ASC group. See Figure 4 and Table 3. Correlational analysis showed that IQ was not significantly correlated with scores on these additional self-referential measures (see Additional file 1: Figure S2). This suggests that extreme scores observed in AG cannot be explained simply by lower IQ in AG.

**Table 3:** **Additional self-referential measures**^**a**^

	**Control**	**ASC**	**AG**
PSCS	30.50 (28.80 to 31.77)	29.80 (27.87 to 31.67)	24
SFA	0.11 (0.0987 to 0.1207)	0.09 (0.0817 to 0.1050)	0.11
TAS	41.97 (38.67 to 45.13)	58.37 (53.37 to 63.33)	70
DIF	13.50 (11.87 to 15.27)	20.03 (17.77 to 22.50)	24
DDF	11.10 (9.47 to 12.90)	16.87 (14.87 to 18.80)	20
EOT	17.37 (15.97 to 18.90)	21.47 (19.67 to 23.10)	26

^a^Mean and 95% bootstrap bias-corrected and accelerated confidence intervals (in parentheses) are given for the control and ASC groups. AG’s performance is given in the third column. On each measure except for SFA, AG’s performance was more extreme than the 95% confidence intervals for both control and ASC groups. ASC, Autism Spectrum Conditions; PSCS, Private Self-Consciousness Scale; SFA, implicit index of self-focused attention based on the percentage of first-person pronouns used in the Self-Focus Sentence Completion task; TAS, Toronto Alexithymia Scale; DIF, Difficulty Identifying Feelings subscale of TAS-20; DDF, Difficulty Describing Feelings subscale of the TAS-20; EOT, Externally Oriented Thinking subscale from the TAS-20.

**Figure 5:**
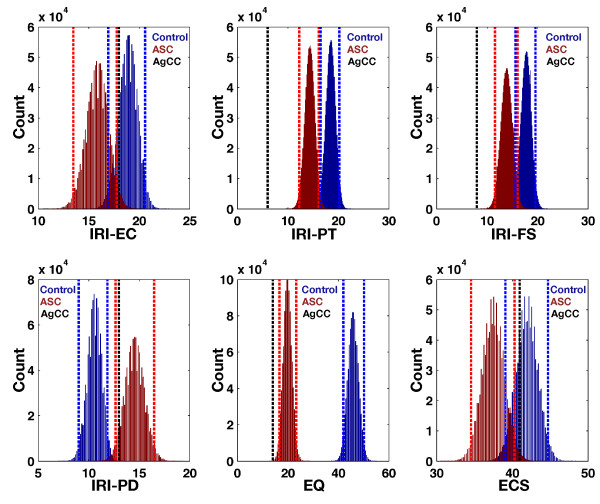
**Additional social-cognitive measures.** This figure plots the bootstrap distributions of the mean along with the 95% bias-corrected and accelerated bootstrap confidence intervals (vertical dotted lines). The ASC group is depicted in red and the control group in blue. AG is depicted as the black dotted line. Abbreviations: IRI-EC, Empathic Concern subscale from the IRI; IRI-PT, Perspective Taking subscale of the IRI; IRI-FS, Fantasy subscale of the IRI; IRI-PD, Personal Distress subscale of the IRI; EQ, Empathy Quotient; ECS, Emotional Contagion Scale; ASC, autism spectrum condition; AgCC, agenesis of the corpus callosum.

**Figure 6:**
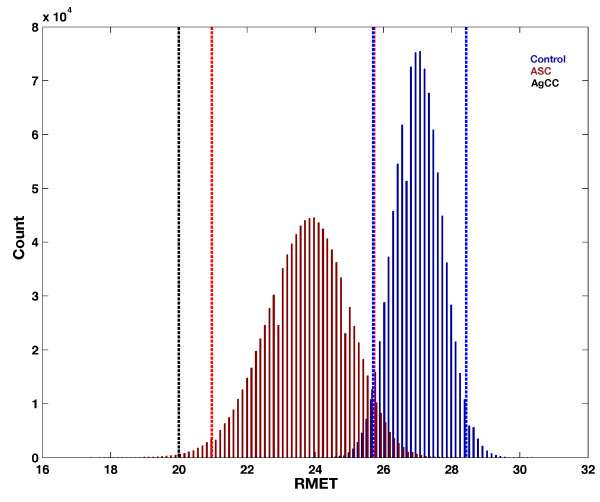
**Reading the mind in the eyes test (RMET) performance.** This figure plots the bootstrap distribution of the mean on the Reading the Mind in the Eyes test (RMET) along with the 95% bias-corrected and accelerated bootstrap confidence intervals (vertical dotted lines). The ASC group is depicted in red and the control group in blue. AG is depicted as the black dotted line. Abbreviations: RMET, Reading the Mind in the Eyes test; ASC, autism spectrum condition; AgCC, agenesis of the corpus callosum.

On additional social-cognitive measures, AG endorsed lower levels of empathy on the EQ and lower scores on the IRI perspective-taking and fantasy subscales compared to Control and ASC groups. AG also performed more poorly on the RMET task than both groups. In contrast, on the ECS and IRI-EC, AG self-reported significantly higher scores than the ASC group, but was within the confidence intervals for the control group. For IRI-PD, AG’s score was within the confidence intervals for the ASC group. See Figures 5 and 6 and Table 4. Correlational analysis showed that VIQ was not significantly correlated with any of these additional social-cognitive measures except the RMET. On the RMET, VIQ positively correlated with performance (*r* = 0.5988, *P* = 0.001) (see Additional file 1: Figure S2). This suggests that extreme performance on the RMET by AG could be partially explained by lower IQ. However, IQ could not explain extreme scores observed on other measures.

**Table 4:** **Additional social-cognitive measures**^**a**^

	**Control**	**ASC**	**AG**
IRI-EC	18.93 (16.93 to 20.60)	15.83 (13.47 to 17.80)	18
IRI-PT	18.50 (16.50 to 20.23)	14.33 (12.27 to 16.13)	6
IRI-FS	17.77 (15.63 to 19.63)	13.87 (11.60 to 16.07)	8
IRI-PD	10.60 (9.03 to 11.87)	14.53 (12.67 to 16.50)	13
EQ	43.93 (41.97 to 50.17)	19.83 (16.63 to 23.33)	14
RMET	27.03 (25.70 to 28.43)	23.73 (20.97 to 25.73)	20
ECS	41.97 (39.10 to 44.73)	37.47 (34.57 to 40.30)	41

^a^Mean and 95% bootstrap bias-corrected and accelerated confidence intervals (in parentheses) are given for the control and ASC groups. AG’s performance is given in the third column. On each measure except for ECS, IRI-EC, and IRI-PD, AG’s performance was more extreme than the 95% confidence intervals for both control and ASC groups. ASC, Autism Spectrum Condition; IRI-EC, Empathic Concern subscale from the IRI; IRI-PT, Perspective Taking subscale of the IRI; IRI-FS, Fantasy subscale of the IRI; IRI-PD, Personal Distress subscale of the IRI; EQ, Empathy Quotient; RMET, Reading the Mind in the Eyes Test; ECS, Emotion Contagion Scale.

## Discussion

In this study, we examined a patient with diagnoses of both ASC and AgCC on a range of self-referential and social-cognitive measures known to be atypical in ASC. As a potential neuroanatomical model for ASC, we followed the idea that AgCC might be a potential extreme of smaller CC volume consistently observed in ASC [12]. Thus, within social-cognitive and self-referential domains where there is some prior work showing potential abnormalities in both ASC and AgCC [10,35,48,52-54,66,70], we tested the idea that if the CC is critical in these domains, this patient with AgCC and ASC would show more extreme performance than that observed in both the ASC and control comparison groups. We found that across most measures in both domains, this patient with AgCC and ASC was atypical, even with respect to the ASC comparison group, in that deficits were more pronounced than what is typically observed on average in ASC.

Within the SRE paradigm, the ASC comparison group showed reduced memory sensitivity compared to controls specifically in the Self and Friend conditions [35]. In contrast, AG exhibited worse memory sensitivity compared to both comparison groups across all conditions. This pattern is partially suggestive of a general memory deficit that occurs across all levels of processing, from very deep encoding during self-referential processing to shallow encoding of information based on linguistic characteristics (i.e. counting syllables). However, the biggest differences were observed in social-cognitive conditions where memory was examined in relation to other people (i.e. Friend and Potter). This potentially suggests that while memory performance is generally affected, the areas that are hit hardest are social-cognitive information processing. Alternatively, part of the general trend for reduced memory across all conditions may be explained by AG’s lower IQ. VIQ was positively correlated with memory sensitivity in the ASC group for both Friend and Potter conditions. Given that AG also had a diagnosis of ASC, it is reasonable to infer from this result that a partial explanation for the reduced memory in these conditions could be because of lower IQ. However, it is also possible that IQ is not the only reason for decreased memory performance. Some variability in AG’s decreased performance is likely to be due to true effects in the domains of self-referential and social cognition. This is suggested by the lack of correlations with IQ observed on other self-referential and social-cognitive measures where AG also shows more extreme scores.

We also evaluated whether AG reaps cognitive benefits from self-referential information processing by comparing self-memory sensitivity to memory sensitivity across non-self conditions. Here we computed the difference-score between self-memory sensitivity and non-self-memory sensitivity, and increasingly positive scores indicate more benefit for self-referential information processing. Since we are dealing with groups that also have deficits in the social domain, using social-cognitive conditions such as Friend and Potter as the comparison to self-memory sensitivity is not ideal, because pronounced social-cognitive deficits can affect this memory sensitivity difference-score. This can clearly be seen in the difference-scores for Self-Friend and Self-Potter. Here AG appears to benefit substantially from self-referential information processing. However, this interpretation should be avoided, as the effects are driven primarily by the substantial drop-off in memory sensitivity for the Friend and Potter conditions in AG. Given these arguments, the most unambiguous measure for assessing whether AG reaps cognitive benefits of self-referential information processing is the Self-Syllable difference-score. Here memory sensitivity in the Syllable condition shows relatively less drop-off, and the difference-score cannot be confounded by additional deficits in social-cognitive processing. Here we find that AG benefits less from self-referential processing, as indexed by a lower Self-Syllable memory sensitivity difference-score compared to the control group. AG cannot be considered as an extreme from the ASC group though, because this Self-Syllable memory sensitivity difference-score was similar to what was observed in the ASC group.

Similar patterns of more extreme scores from AG were apparent in the self-referential domain on the PSCS, TAS, and subscales of the TAS and in the social-cognitive domain on the IRI-PT, IRI-FS, RMET, and EQ. Of these measures, only RMET correlated with IQ, suggesting that the extreme profile of scores from AG is relatively independent of his lower IQ. For the RMET, it may be that once IQ effects are accounted for, AG’s performance may not necessarily be at the extreme of the performance of the ASC group. In prior work, it is noteworthy that, in a larger sample of individuals with AgCC, there was overlap between the AgCC scores with both the control and ASC distributions on the RMET and no evidence of extreme deficits compared to the ASC group was observed [25]. However, the overall conjunction of extreme scores observed on both the SRE paradigm and across various other self-referential and social-cognitive measures suggests that AG’s performance represents an extreme with respect to what is observed on average in ASC. Thus, rather than AG exhibiting performance typical of ASC without AgCC, the current set of results is compatible with the idea that additional abnormalities in the CC in ASC may be relevant for understanding degree of self-referential and social-cognitive difficulties in ASC. To this end, focusing on cases with AgCC and a diagnosis of ASC may be a good neuroanatomical model for parsing the critical role that normative callosal development may have on these domains.

It should also be noted where AG was not an extreme with respect to the comparison groups. We found that on IRI-EC, IRI-PD, and ECS that AG’s scores were more similar to the control group. All of these measures share one commonality in being affective in nature. This suggests that the CC may not be as critical for understanding aspects of empathy difficulties in ASC that are affective in nature. This result stands in contrast to subscales measuring more cognitive aspects of empathy (i.e. perspective taking and fantasy subscales of the IRI), where AG was more extreme than both comparison groups. This may be suggestive that AgCC affects high-level cognitive components of social cognition while leaving affective components relatively spared. Within the self-referential domain, AG was also similar to the control group on the implicit measure of SFA that measures first person pronoun usage under conditions where the participant is primed to use self-references. While AG’s scores were significantly different from that observed in the ASC comparison group, the directionality is in keeping with a prior report showing that first person pronoun usage in narratives was increased in AgCC [46].

This apparent preservation of function in specific domains may be suggestive of two possibilities. First, there may be a certain degree of redundancy built into the neural architecture for affective function and pronoun usage, such that lacking large parts of anterior callosal regions do not have a major impact on these domains. Second, the developmental nature of AgCC might allow for appropriate compensatory mechanisms to develop over time in these domains. Future research could compare these results with that from patients who have an acquired white matter lesion in the anterior callosal region later in life. Finally, with respect to the self-reported affective empathy measures that appear to be spared, it remains to be seen whether AgCC patients would show similar spared function in the domain of affective empathy on performance-based tests. It may be that affective empathy is affected in AgCC, but that these patient’s self-referential difficulties obscure accurate measurements of their own affective empathy abilities.

There are several caveats and limitations to highlight about the current study. First, analyses showed that on some measures, variation related to IQ may be important. Given that our case (AG) had IQ scores that were lower than both comparison groups, it remains to be seen how well the results here are generalizable in studies where AgCC and comparison groups are matched on IQ. Our suggestion is that the role of IQ may be limited for interpreting the results here, as we found many pieces of evidence where AG’s performance was more extreme than the comparison groups and IQ was not generally correlated with performance. Second, this case report may not be fully generalizable to all patients with concurrent AgCC and ASC. It will be important for future work to expand on these observations with larger prospective work. Third, the current results are restricted to self-report measures and constrained experimental tasks involving situations likely not present during everyday functioning and real-world scenarios. Therefore, it remains to be seen whether the inferences from this study would bear out in future work using more naturalistic measures in real-world social situations. Fourth, the current work assesses the question about corpus callosum variation and autism from a categorical point of view. For example, in this study groups are defined categorically by their clinical diagnosis and within autism, the patient stands out as categorically distinct at the level of the corpus callosum. This is not the only way to investigate this question. Another way to address these types of questions is to look at the phenomenon from a continuous perspective where both autism and the corpus callosum are measured on a spectrum. This approach is intriguing and should be followed up in future work. This work sets up the hypothesis that from a dimensional perspective, better performance in areas of self-referential and social cognition might be related to continuous variation in CC size.

Finally, it is important to note the nature of AG’s callosal development. Rather than lacking a CC altogether, AG did have intact connections within the posterior section of the CC. At present there is no clear indication of whether partial versus complete AgCC cases are different with respect to performance in self-referential and social-cognitive domains. A recent MEG study found no differences between partial and complete AgCC with respect to global connectivity across alpha, gamma and beta bands [100]. Another recent study of fMRI resting state connectivity found strikingly similarities between AgCC and those with an intact CC. The exceptions to this were reduced connectivity in the posterior midline areas typically connected via the posterior sections of the CC [101]. Thus, because it has been postulated that these posterior midline areas may be important for self-referential and social-cognitive development [102], it is possible that AG’s partial connections make him a less extreme case than would be seen in complete AgCC. However, this remains open for future research on the topic.

## Conclusions

In conclusion, we report that a case of partial AgCC and ASC showed more extreme scores than those typically found on measures that elicit group-differences in self-referential and social cognition in ASC compared to neurotypical controls. These results confirm prior findings suggesting that individuals with AgCC have marked problems in these domains. Furthermore, the current work suggests potential in viewing AgCC as a neuroanatomical model associated with ASC that may allow us to gain further insight into the mechanisms that may be important for understanding difficulties in self-referential and social cognition.

The MRC AIMS Consortium is a UK collaboration between the Institute of Psychiatry at Kings College, London, the Autism Research Centre and Brain Mapping Unit at the University of Cambridge, and the Autism Research Group at the University of Oxford. The Consortium members are, in alphabetical order, Bailey AJ, Baron-Cohen S, Bolton PF, Bullmore ET, Carrington S, Chakrabarti B, Daly EM, Deoni SC, Ecker C, Happé F, Henty J, Jezzard P, Johnston P, Jones DK, Lai, MC, Lombardo MV, Madden A, Mullins D, Murphy C, Murphy DG, Pasco G, Sadek SA, Spain D, Stewart R, Suckling J, Wheelwright S, Williams SC.

## Abbreviations

ADI–R: Autism Diagnostic Interview–Revised; ADOS: Autism Diagnostic Observation Schedule; AgCC: Agenesis of the corpus callosum; AG: Patient with partial agenesis of the corpus callosum and a clinical diagnosis of Asperger syndrome; AQ: Autism spectrum quotient; AS: Asperger syndrome; ASC: Autism spectrum condition; CC: Corpus callosum; d′: Index of memory sensitivity; DDF: Difficulty Describing Feelings subscale of the TAS; DIF: Difficulty Indentifying Feelings subscale of the TAS; EOT: Externally Oriented Thinking subscale of the TAS; EQ: Empathy quotient; HFA: High-functioning autism; IRI: Interpersonal Reactivity Index; IRI-PT: Perspective Taking subscale of the IRI; IRI-EC: Empathic Concern subscale of the IRI; IRI-PD: Personal Distress subscale of the IRI; IRI-FS: Fantasy subscale of the IRI; ToM: Theory of mind; MRI: Magnetic resonance imaging; PSCS: Private Self-Consciousness Scale; RMET: Reading the Mind in the Eyes test; SFA: Implicit measure of self-focused attention index by the percentage of first-person pronouns used in the Self-Focus Sentence Completion test; SRE: Self-reference effect in memory paradigm; TAS: Toronto Alexithymia Scale; VIQ: Verbal IQ; WASI: Wechsler Abbreviated Scale of Intelligence.

## Competing interest

The authors declare they have no competing interest.

## Authors’ contributions

MVL conceived and carried out the study, ran all statistical analyses, and drafted the manuscript. BC, MCL, MRC AIMS Consortium, and SBC contributed to study design. BC, MCL, and SBC helped draft the manuscript. SBC and the MRC AIMS Consortium obtained funding and coordinated the study. All authors read and approved the final manuscript.

## Supplementary Material

Additional file 1 **Figure S1.** Reaction times on SRE paradigm. **Figure S2.** Correlations between IQ scales and dependent variables within the ASC group. Click here for file
