# Supplementary material for: Self-referential and social cognition in a case of autism and agenesis of the corpus callosum
Source: Mol Autism. 2012 Nov 21;3:14. doi: 10.1186/2040-2392-3-14 (PMC3522057; doi:10.1186/2040-2392-3-14)
Supplement: Additional file 1 — Figure S1. Reaction times on SRE paradigm. Figure S2. Correlations between IQ scales and dependent variables within the ASC group. [file 2040-2392-3-14-S1.doc]

**Supplementary Figure 1: Reaction times on SRE paradigm**

*This figure plots the correlations between IQ and the multiple dependent variables assessed in this study within the autism spectrum conditions (ASC) group. Correlations are estimated with robust regression in order to be insensitive to outlying data points.*

*Abbreviations: VIQ, verbal IQ; PIQ, performance IQ; FIQ, full scale IQ; d’, memory sensitivity; PSCS, Private Self-Consciousness Scale; SFA, self-focused attention index measured as the percentage of first person pronouns used in the Self-Focus Sentence Completion Task; TAS, Toronto Alexithymia Scale; DIF, Difficulty Identifying Feelings subscale of the TAS-20; DDF, Difficulty Describing Feelings subscale of the TAS-20; EOT, Externally Oriented Thinking subscale from the TAS-20; IRI-EC, Empathic Concern subscale from the IRI; IRI-PT, Perspective Taking subscale of the IRI; IRI-FS, Fantasy subscale of the IRI; IRI-PD, Personal Distress subscale of the IRI; EQ, Empathy Quotient; ECS, Emotional Contagion Scale.*

**Supplementary Figure 2: Correlations between IQ scales and dependent variables within the ASC group.**

*This figure plots the correlations between IQ and the multiple dependent variables assessed in this study within the autism spectrum conditions (ASC) group. Correlations are estimated with robust regression in order to be insensitive to outlying data points.*

*Abbreviations: VIQ, verbal IQ; PIQ, performance IQ; FIQ, full scale IQ; d’, memory sensitivity; PSCS, Private Self-Consciousness Scale; SFA, self-focused attention index measured as the percentage of first person pronouns used in the Self-Focus Sentence Completion Task; TAS, Toronto Alexithymia Scale; DIF, Difficulty Identifying Feelings subscale of the TAS-20; DDF, Difficulty Describing Feelings subscale of the TAS-20; EOT, Externally Oriented Thinking subscale from the TAS-20; IRI-EC, Empathic Concern subscale from the IRI; IRI-PT, Perspective Taking subscale of the IRI; IRI-FS, Fantasy subscale of the IRI; IRI-PD, Personal Distress subscale of the IRI; EQ, Empathy Quotient; ECS, Emotional Contagion Scale.*
